# Sustainable Development of Enhanced Luminescence Polymer-Carbon Dots Composite Film for Rapid Cd^2+^ Removal from Wastewater

**DOI:** 10.3390/molecules25153541

**Published:** 2020-08-03

**Authors:** Mohammed Abdullah Issa, Zurina Z. Abidin

**Affiliations:** Department of Chemical and Environmental Engineering, Faculty of Engineering, University Putra Malaysia, Serdang 43400 UPM, Selangor, Malaysia

**Keywords:** biomass waste, *N* doped carbon dots, adsorption, Cd (II), mechanism, water remedy

## Abstract

As a remedy for environmental pollution, a versatile synthetic approach has been developed to prepare polyvinyl alcohol (PVA)/nitrogen-doped carbon dots (CDs) composite film (PVA-CDs) for removal of toxic cadmium ions. The CDs were first synthesized using carboxymethylcellulose (CMC) of oil palms empty fruit bunch wastes with the addition of polyethyleneimine (PEI) and then the CDs were embedded with PVA. The PVA-CDs film possess synergistic functionalities through increasing the content of hydrogen bonds for chemisorption compared to the pure CDs. Optical analysis of PVA-CDs film was performed by ultraviolet-visible and fluorescence spectroscopy. Compared to the pure CDs, the solid-state PVA-CDs displayed a bright blue color with a quantum yield (QY) of 47%; they possess excitation-independent emission and a higher Cd^2+^ removal efficiency of 91.1%. The equilibrium state was achieved within 10 min. It was found that adsorption data fit well with the pseudo-second-order kinetic and Langmuir isotherm models. The maximum adsorption uptake was 113.6 mg g^−1^ at an optimal pH of 7. Desorption experiments showhe that adsorbent can be reused fruitfully for five adsorption-desorption cycles using 0.1 HCl elution. The film was successfully applied to real water samples with a removal efficiency of 95.34% and 90.9% for tap and drinking water, respectively. The fabricated membrane is biodegradable and its preparation follows an ecofriendly green route.

## 1. Introduction

Water is the prime component of human survival. Nevertheless, environmental pollution caused by heavy metals owing to the inappropriate disposal of wastewater is becoming a worldwide concern [1,2]. Cd(II) metal is one of the most toxic substances that tend to accumulate in living organisms. According to the Disease Registry and Environmental Protection Agency (EPA), Cd(II) ranks seventh on the top 20 toxic chemicals priority list [3]. Long-term exposure to Cd(II) results in a series of harmful diseases in humans ecosystem damage [4]. Due to its toxic effects on human health, the separation and elimination of Cd^2+^ from aquatic media is still a hot research topic. Among the solutions, zeolites [5], polymeric compounds [6] and carbon-based materials [7,8] are the most widely used sorbents for the removal of chemical pollutants, due to their large surface area and tunable pore size.

Nowadays, carbon dots (CDs) have gained considerable attention for heavy metal removal from aqueous systems [9,10]. These CDs, with sizes less than 10 nm, exhibit outstanding properties like ease of preparation, non-toxic nature and excellent luminescence behavior. These features make CDs ideal to be applied for wastewater remediation, photocatalysis, cell imaging, cancer therapy and metal sensing [11,12,13,14]. Many protocols including an arc-discharge approach [15], electrochemical oxidation [16], microwave treatment [17] and a hydrothermal route [18] have been demonstrated for the production of CDs. Among all the reported protocols, the hydrothermal route is the most preferred technique for its simplicity, mild conditions and high fluorescence quantum yield (QY).

More recently, hydrothermal approaches using a variety of natural carbonaceous precursors have been successfully employed to obtain CDs [19,20,21,22,23,24]. In particular, the usage of lignocellulosic wastes was fruitfully utilized for their cost-effective and eco-friendly resources [25,26,27,28,29,30,31]. However, existing works have indicated several drawbacks like weak QY and harsh synthesis conditions [27,28]. One should note that the replacement and clustering degree of cellulose rings and the chain length, as well as the choice of additives, are the key factors influencing the final QY of CDs. Carboxymethylcellulose (CMC) is the main modification of cellulose in which carboxymethyl species replace several hydrogen atoms of the hydroxyl moieties on the cellulose framework [10,32]. These biocompatible resources enriched with ether and hydroxyl groups can accelerate the synthesis process through successive substitution reactions and enhance the removal uptake by providing adsorption sites [2,29]. Thus, full exploitation of these low-value wastes to recycle them and develop new value-added carbonaceous materials is of great interest. The encapsulation of CDs into solid-state film is still challenging as they show photo-quenching properties after the immobilization processes, make them unsuitable for application in a variety of fields [4].

Previous works have reported the workability of incorporating CDs with metallic compounds or polymers without affecting the optical performance [33,34]. There have been a few works explaining the integration of CDs into hydrogels for several functions. Among them, polyvinyl alcohol-based films were frequently used to prepare freestanding luminescence films for green CD encapsulation [35,36,37,38], due to their biocompatibility, non-toxicity and hydrophilicity [39,40,41,42,43]. Despite the strong optical efficiency that was achieved using CDs-based hybrid films, several existing drawbacks, including long equilibration times of more than 1 h [7,44,45] and low removal uptake [45,46,47], have restricted their Cd^2+^ removal applications. Therefore, it would be sensible to develop highly photoluminescence CDs to enhance their performance towards heavy metal removal from contaminated water. It is well known that surface doping of CDs with N can effectively tune its electronic structure, provides available sites and enhanced PL, which are great significance for efficient and selective recognition and capture of metal ions [10,48].

In this regard, herein, we report a versatile one-pot route for production CDs from CMC with the assistance of PEI dopant. The as-obtained CDs were then integrated with a PVA matrix to develop a (PVA-CDs) free-standing membrane, which was further applied for Cd^2+^ removal from wastewater. The physicochemical properties of bare CDs and PVA-CDs film were thoroughly studied using different analytical techniques including low and high-resolution transmission electron microscopy (TEM, HRTEM), X-ray diffraction (XRD), zeta potential (η), X-ray photoelectron spectra (XPS), Fourier transform infrared (FTIR) and UV/VIS spectroscopy (UV–Vis) and fluorescence spectra (PL). Impressively, the as-developed PVA-CDs film exhibited blue emission with enhanced QY up to 47% in comparison to the isolated CDs (greenish, 44%), which could be ascribed to a H bonding effect. The photo- and thermostability of the film resulted in no apparent reduction in the PL emission. The developed film was utilized for Cd^2+^ removal from water and emphasis was placed on the adsorption mechanism by studying the sorption kinetics and isotherms. Furthermore, desorption and regeneration studies were also conducted to evaluate the reusability of the film.

## 2. Results and Discussion

### 2.1. Synthesis of CDs and PVA-CDs

**Figure 1:**
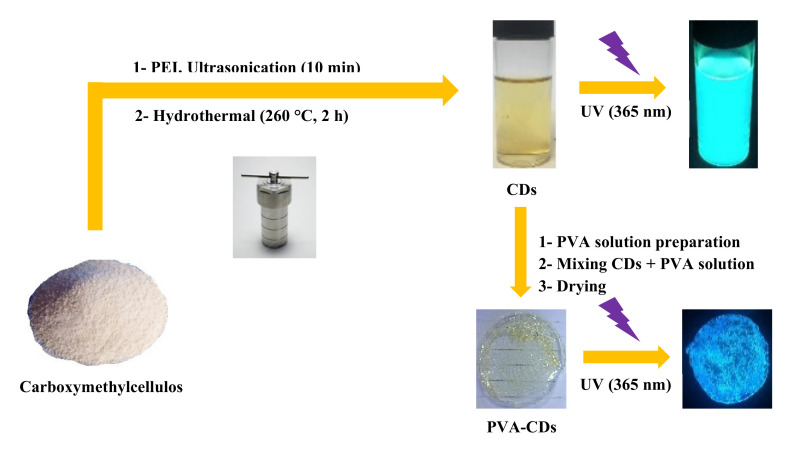
Schematic representation of the preparation of CDs and PVA-CDs film from CMC waste.

Figure 1 shows a schematic representation of CDs and PVA-CDs composite development. CMC, as a green and economic resource, is used as a carbon precursor for the production of fluorescent CDs. In contrast to other lignocellulosic materials reported in the literature [27,29], CMC of empty fruit bunches is enriched with ether and hydroxyl moieties which could play a major role in the enhancement of substitution degree and carbonization acceleration. Branched PEI was selected as a dopant for its feature to inject electron-rich N into the final framework of CDs and increasing QY with the ability to capture metal ions [9]. The as-formed CDs exhibit a yellow color, showing greenish emission under UV-lamp (365 nm), good water solubility and long shelf-life (up to 1 year) with no apparent agglomeration. These CDs were encapsulated with PVA polymer host to develop a solid-state hybrid film for heavy metal complexation in an attempt to overcome the separation issues of the liquid phase [4]. In comparison to the liquid CDs, the as-obtained film is transparent under daylight while they exhibited a bright blue emission under UV exposure. This transformation from greenish to blue could be due to the formation of hydrogen bonds from the numerous interactions of CD surface sites and the PVA substrate [41,49].

### 2.2. Physical Structure

**Figure 2:**
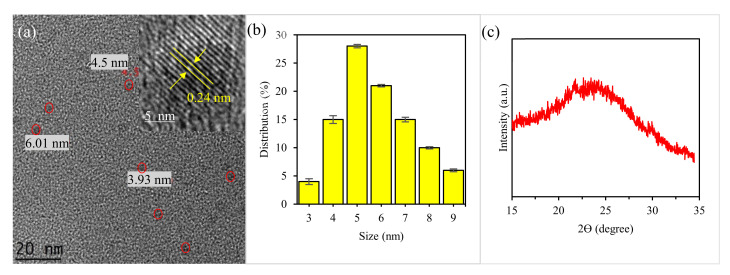
(**a**) TEM of CDs, (**b**) particle size distribution, (**c**) XRD patterns of CDs. Inset: HRTEM of CDs.

Figure 2 show the TEM, HRTEM and XRD results of the CDs. TEM images (Figure 2a) reveal that the obtained CDs have a monodispersed spherical structure, with an average size of 4.1 nm. Based on the HRTEM images (inset of Figure 2a), these nanodots have high crystallinity domains, demonstrating the presence of a graphitic inner core. The well-resolved lattice fringes show an interplanar distance of 0.24 nm, which is close to the (100) facet of graphite, similar to previous reported studies [50,51,52]. Based on the particle size distribution histogram, which was calculated from one hundred nanoparticles (Figure 2b), the CDs have a size distribution ranging from 3 to 9 nm with an average diameter of about 4.2 ± 0.5 nm. The XRD data displayed a diffraction peak at a 2*Ɵ* value of 23.8° attributed to the (002) lattice spacing of graphitic-like carbon, in line with other works [50,51,52,53]. The measured zeta potential of the CDs (Figure S1) found to be −8.71 mV at pH 7, implying a negatively-charged CDs edges.

### 2.3. Chemical Structure

**Figure 3:**
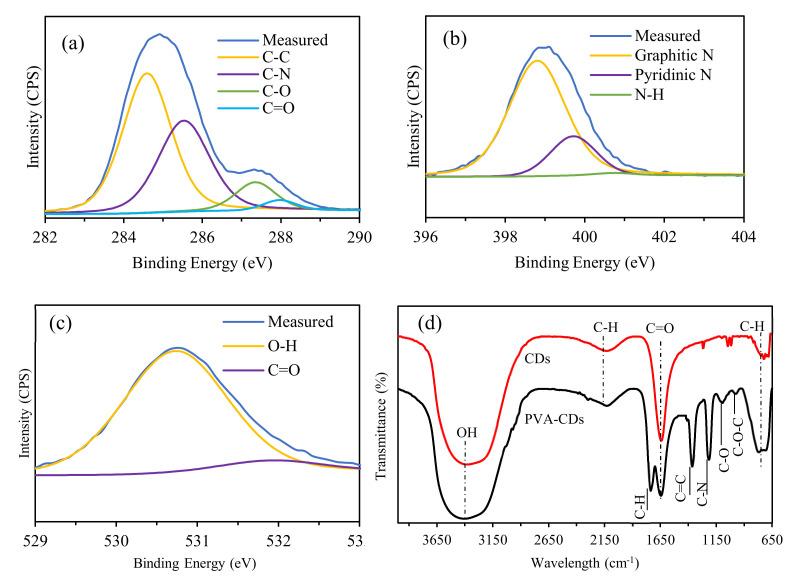
The high-resolution XPS survey of (**a**) C1s, (**b**) N1s and (**c**) O1s, (**d**) FTIR spectra of CDs and PVA-CDs.

XPS was used to gain insight into the chemical composition of the used and formed materials. As presented in Figure S2, the XPS spectrum of CMC is mainly composed of C1s and O1s. Meanwhile, CDs indicated the presence of C1s, O1s and N1s, suggesting the formation of graphene CDs with the abundance of N. It can also be seen that the peaks of both C1s and O1s were remarkably higher than that of CMC, implying the occurrence of aromatization reaction with a high degree of surface oxidation. The high-resolution XPS survey of C1s (Figure 3a) displayed C-C, C-N, C-O and C=O at 284.6, 285.5, 287.4, and 287.9 eV, respectively. The dominant peak of sp^2^ hybridized C-C (52.4%) suggests a high degree of graphitization. The N_1_s band (Figure 3b) showed three peaks at 398.8, 399.7 and 400.8 eV associated with graphitic N, pyridinic N and N–H, respectively. The formation of graphitic and pyridinic N indicate the effective N-doping of CD. Meanwhile, non-graphitic amines like N-H distributed around CDs through Maillard chemistry reaction [54,55,56,57,58,59,60]. The O_1_s spectrum (Figure 3c) showed two peaks at 530.7 and 531.9 eV corresponding to O-H and C=O, respectively. It is worth noting that the incorporation of active moietises including N-H and C=O can effectively enhance the QY of CDs, which provide more active sites for capturing a variety of analytes [4,13,48].

The corresponding elemental compositions (in atomic ratios) are summarized Table S1. The atomic ratio C/N/O is found to be 67/12/21, demonstrating the successful doping of N-atom into the conjugated carbon skeleton domains of the CDs [61]. The high content of carbon compared to oxygen emphasizes a fruitful aromatization reaction with the elimination of O moieties. These active sites can tune the electronic structure, surface chemical characteristics and PL emission, which are vitally necessary for the selective sensing and removal of different metal ions [10,25].

Further confirmation about the chemical binding and surface functionalities was obtained using FTIR as presented in Figure 3d. In comparison to the spectra of CDs, new peaks at 1438 and 1738 cm^−1^ were formed, which are ascribed to hybridized C=C and C-H moieties, respectively [40]. Besides, there is a shift from 1266 to 1368 cm^−1^ and an increase in the intensity of the C-N band, implying a plausible involvement of graphitic N in the encapsulation process. Furthermore, the change in position and shape of the C–H bending from 725 to 772 cm^−1^ indicates the formation of hydrogen bonds between CDs and PVA polymer [40]. Moreover, an obvious increment and shift of the C-O, C-O-C and C=O bands from 1046 to 1096 cm^−1^, 1017 to 979 cm^−1^ and 1641 cm^−1^ to 1647 cm^−1^, respectively, were also observed. This provides evidence that both O species and topological traps were altered [49,62]. The above results are consistent with the XPS data, confirming the successful interaction between PVA host and CDs.

According to the XPS and FTIR data, it can be said that the abundance of H-containing functional groups on the surface of CDs plays a significant role to interact covalently with the PVA matrix [63]. In details, the presence of –OH, –COOH, and –NH implies that the H-containing groups are partly tethered on the edge of the CDs, rather than being completely encapsulated in the CDs [63]. From the FTIR spectra in Figure 3d, the appearance and enhancement of the C=C, C-N and C-H bands demonstrate the existence of chemical linkages between the CDs with the PVA host, forming a cross-linking network domain.

### 2.4. Optical Properties

UV-vis absorption and PL spectra were recorded to study the optical behavior of the liquid and solid-state CDs. The UV-Vis absorption spectra (Figure 3a) of both CDs and PVA-CDs film exhibit two absorption peaks centered at 294 and 340 nm, corresponding to the π- π^*^ transition and n- π^*^ transition of C=C and C=O, in line with previously reported data for carbon dot-based materials [10,40].

The PL spectra (Figure 3b) reveal that the excitation/emission maxima of PVA-CDs film were red-shifted by about 10 and 3 nm, respectively, compared to that of CDs. This may be due to the PVA environment, which can act as a surface passivation agent for CDs [64]. The QY of the developed PVA-CDs composite film was determined and found to be 47%, which is higher than that of CDs (inset of Figure 2b). The QY enhancement might be due to hydrogen bonding interactions in the PVA/CDs composite as suggested by the FTIR analysis (Figure 3b). This binding can produce plenty of physical crosslinking points for the CDs in the more confined environment of the PVA matrix, which provide a stabilization effect on the electron/hole pairs for more efficient radiative recombination, as reported in the literature [41,49,64]. The emission spectra (310–380 nm in range) of the CDs remained unshifted as the excitation wavelength was varied from 310–350 nm. Beyond 350 nm, a red-shift of the PL intensity was observed. The particle size selection and surface traps of CDs are the main factors responsible for the emission variation [27,51,65,66]. In contrast to the liquid CDs, PVA-CDs film exhibits only excitation-independent emission phenomena when exposed to varied excitation wavelengths (Figure 7c), which agrees with previous work [40]. This phenomenon is more favorable for a variety of applications as it prevents auto-luminescence [67].

To evaluate the photostability of the PVA-CDs composite film, the developed films were stored under daylight for one month and the PL spectra were recorded. The PL spectra (Figure S3a) display no apparent loss in PL intensity even after this duration of daylight illumination, retaining about 97% of their original PL emission. Moreover, the effect of varied temperate on the PL emission was also measured. As presented in Figure S3b, PL intensity retention of 97.8% was shown as the temperature increases from 25 to 65 °C. The enhanced QY and photostability of the PVA-CDs render them ideal for using these films in a wide range of applications including the detection and removal of metal ions.

### 2.5. Heavy Metal Complexation Test Using PVA-CDs Films

XPS and FTIR data confirm that the as-obtained PVA-CDs composite film is enriched with active sites. These sites can act as a bridge for the PVA-CDs to interact with heavy metals leading to metal complexation and rapid removal from wastewater. In this work, the selectivity of the PVA-CDs to adsorb heavy metals was tested in the presence of 50 ppm of Ni^2+^, Pb^2+^, Cd^2+^, Zn^2+^ and Hg^2+^ at pH 7 for 10 min. The results reveal that the removal efficiencies of PVA-CDs film towards Ni^2+^, Pb^2+^, Cd^2+^, Zn^2+^ and Hg^2+^ were about 46.37, 91.26, 61%, respectively (Figure S4). This suggests the more effective chelating kinetics of Cd^2+^ to bind with the functional moieties on the PVA-CDs surface compared to other metal ions [10,68].

### 2.6. Effect of pH and Contact Time on Cd^2+^ Removal

**Figure 4:**
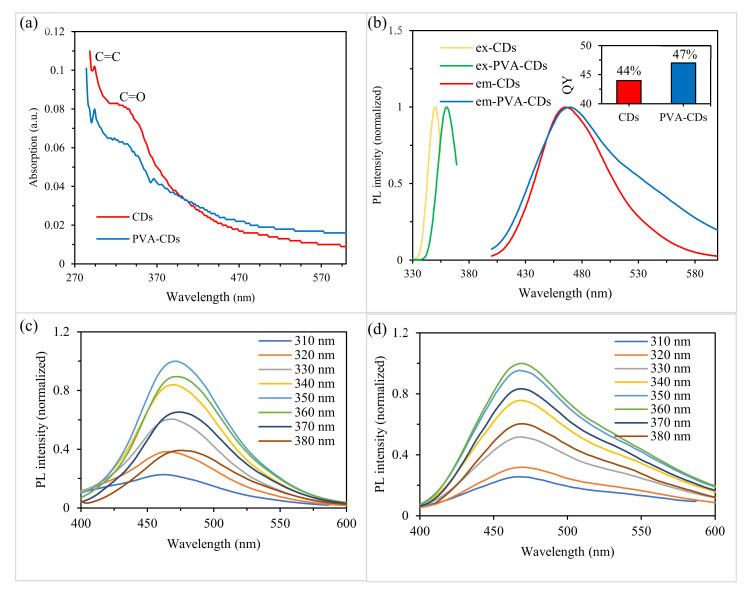
(**a**) UV-Vis absorption spectra of bare CDs and PVA-CDs film with inset photographs under daylight (left) and UV-light (right), (**b**) PL emission with inset showing QY, (**c**) excitation-dependent emission spectra of pared CDs and (**d**) excitation-dependent emission spectra of PVA-CDs film.

To optimize the analytical performance, the influence of pH and response time on Cd^2+^ adsorption using PVA-CDs film were evaluated. Figure 4a shows the % adsorption of Cd^2+^ using PVA-CDs and uncoated CDs at different pH values (3–11). The adsorption efficiency of Cd^2+^ for both PVA-CDs and uncoated CDs were found to rise in alkaline solutions, with a maximum % adsorption of 91 and 83, respectively. The low uptake in acidic medium could be ascribed to the release of H_3_O^+^ ions. The latter compete with Cd^2+^ for the surface sites of the adsorbent, leaving free metal in the suspension [69,70]. However, this effect is reduced with rising pH and the electrostatic attractions become dominant, thus enhancing the adsorption capacity [1]. Next the effect of contact time (from 1 to 25 min) on the % adsorption of Cd^2+^ was studied (Figure 4b). As shown, after only 10 min the PVA-CDs film reached the highest adsorption removal (91%). Meanwhile, an incubation period of 30 min was found sufficient to achieve an adsorption of 86% using uncoated CDs. In comparison to CDs, the PVA-CDs film is more efficient in terms of both adsorption time and removal efficiency. This enhancement in a remarkably short duration could be related to the formation of hydrogen bonds that facilitate provide more electrostatic exchange with Cd^2+^ and thus improve the % adsorption [40].

### 2.7. Adsorption Kinetics and Isotherm

To explain the adsorption of Cd^2+^ onto PVA-CDs, the first- and second-order kinetic models were evaluated, which can be described by the following equations:

First-order kinetic model:

(1) $$\log\left(q_{e}-q_{t}\right)=\log q_{e}-\left(k_{1}/2.303\right)t$$

Second-order kinetic model:

(2) $$t/q_{t}=1/\left(k_{2}\;q_{e}^{2}\;\right)+t/q_{e}$$

where *q_t_* and *q_e_* (mg/g) are, respectively, the adsorption capacity at time *t* and equilibrium. *k*_1_ (min^−1^) and *k*_2_ (g (mg min)^−1^) are the rate constants of first- and second-order models, respectively.

The adsorption performance can be determined using Langmuir or Freundlich models using the following equations, respectively:

(3) $$C_{e}/q_{e}=C/q_{m}+1/\left(q_{m\;}k_{L}\;\right)$$

(4) $$\log q_{e}=\log k_{f}+1/n\times \log C_{e}$$

where $q_{e}$ and $C_{e}\;$are the equilibrium adsorption uptake (mg/g) and equilibrium concentration of the metal ions (mg/L), respectively. $q_{m}$ is the maximum adsorption capacity (mg/g) and $K_{L}$ is Langmuir constant. Freundlich variable $K_{f}$ describes the capacity of the absorption experiment and *n* indicates the isotherm nonlinearity.

**Figure 5:**
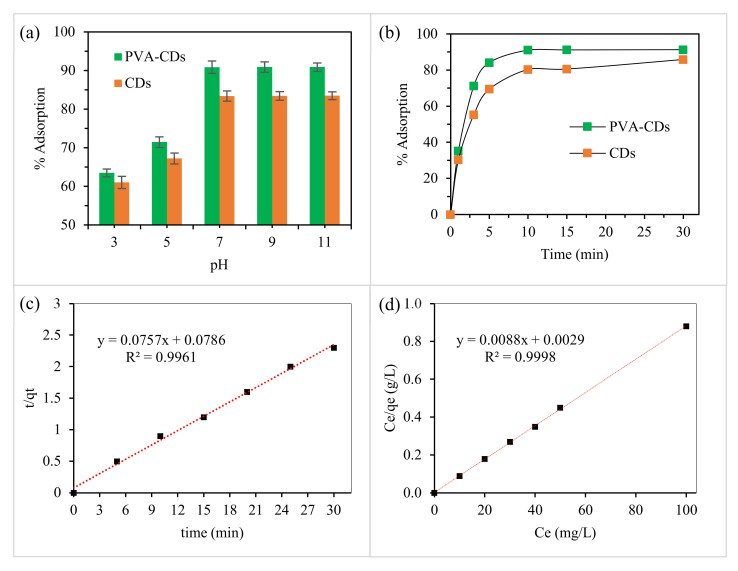
The effect of (**a**) pH and (**b**) contact time (pH = 7) on the adsorption efficiency for Cd^2+^ using CDs and PVA-CDs, (**c**) Pseudo-second-order model for adsorption Cd^2+^ onto PVA-CDs, (**d**) linearity graph of Langmuir isotherm.

Studying the adsorption kinetics is necessary for wastewater treatment as it affords substantial insights into the adsorbent nature and the dynamic process. Thus, the rate of Cd^2+^ removal on PVA-CDs was evaluated from the straight-line plots of ln(*q_e_* − *q_t_*) versus (t) for first-order (Figure 4a) and *t*/*q_t_* against (t) for pseudo-second-order models (Figure 4b). As shown, the pseudo-second-order kinetic model has better correlation parameter values compared to the first-order kinetics (Figure 5c, Figure S5a, Table 1), from which it can be concluded that the mechanism is probably chemisorption [4].

The adsorption isotherm is studied to calculate the adsorption uptake of an adsorbent and also to predict the interaction process. For instance, the Langmuir model proposes that the adsorption occurs by monolayer formation with no remarkable interaction between the adsorbent and adsorbate moieties, whereas Freundlich assumed that the adsorption capacity occurs on a heterogonous adsorbent surface [69]. In this regard, the adsorption isotherms (Figure 5d and Figure S5b) under the optimal conditions at room temperature were plotted. As presented in Table 1, it can be seen that Langmuir isotherm was a better fit than the Freundlich model according to the correlation coefficients (R^2^). Based on Freundlich model investigation, n values are higher than one, confirming that the sorption reaction could be related to the electrostatic binding between Cd^2+^ and electron-rich species on the PVA-CDs surface [4,71]. The maximum adsorption uptake for cadmium onto N-CDs is determined to be 113.6 mg/g.

To compare the adsorption uptake of N-CDs with other adsorbents, a summary of adsorbent for Cd^2+^ removal is listed in Table 2.

It can be observed that the present nanoprobe has better performance, not only in terms of high adsorption capacity but also of needing less removal time to reach equilibrium. The Langmuir separation factor (RL) was also determined and found to be 0.61, emphasizing the favorable Cd^2+^ adsorption onto PVA-CDs.

### 2.8. Adsorption Mechanism

**Figure 6:**
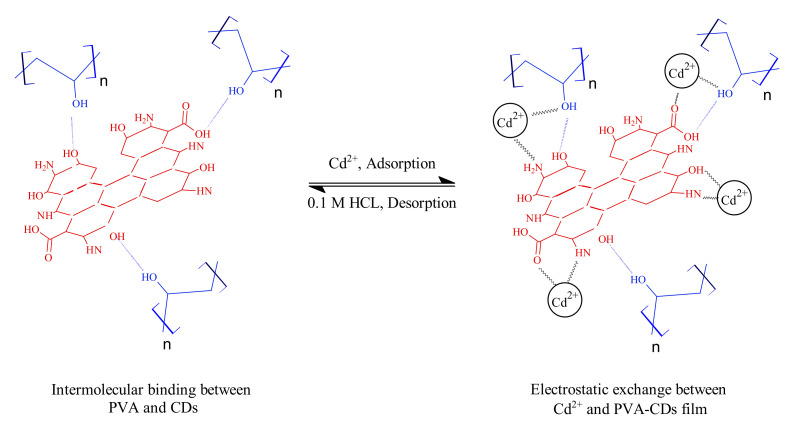
Schematic representation for the adsorption/desorption of Cd^2+^ by PVA-CDs nanocomposite.

Efficiency improvements in heavy metal removal are mainly due to the PVA matrix, as previously reported [10,75,76,77]. Meanwhile, the reduction of Cd^2+^ on uncoated CDs could be explained on the basis of electrostatic attractions between Cd^2+^ centers and electron-rich functionalities around CDs. That means a metal-ligand complex is formed due to the donation effect of electrons from -COO^−^, -OH and -N to the d-orbitals of metal, in line with previously reported works [48,78,79]. However, for the PVA-CDs membrane, a synergetic adsorption activity was noticed. The adsorption/desorption mechanisms of Cd^2+^ ions are illustrated in Figure 6. Recent investigations show that PVA-CDs film favored the rapid adsorption of Cd^2+^ by providing more -OH sites for target metal ions [70,75,76,77]. According to the Pearson assumption, the affinity of heavy metals towards the available sites is ascribed to the soft acid–soft base binding, where the PVA–CDs act as a soft base and Cd^2+^ as a soft acid [4,80].

To support the aforementioned assumption, several analyses were carried out. TEM images and particle size distribution (Figure S6a) show that there is a considerable increase in the nanocomplex size, with an average diameter of 8.6 nm, suggesting the agglomeration of the complex [81,82,83]. In comparison to the uncoated CDs (Figure S1), the surface charges of the hybrid film (Figure S6b) were neutralized with 5.08 eV after adding Cd^2+^ due to the electrostatic effect [48]. FTIR measurements (Figure S6c) prove that there is significant shift of the wavenumbers and increments of the characteristic peaks, particularly C-N, C=O, C-O and C-O-C, demonstrating the involvement of the -COO^−^ and -N-containing groups in Cd^2+^ complexation. All the above observations confirm the complex formation between Cd^2+^ and ligand spices of PVA-CDs.

### 2.9. Desorption and Recyclability

**Figure 7:**
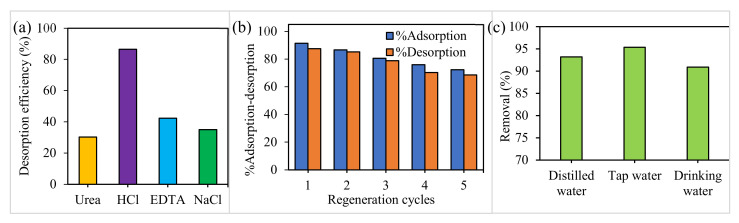
(**a**) Different de-sorbents to recover Cd^2+^ from PVA-CDs, (**b**) reusability study of PVA-CDs for five cycles using HCl and (**c**) real water sample test of PVA-CDs.

The regeneration and reusability performance of the adsorbent was studied to evaluate its activity for practical use. First, recycling experiments to recover the adsorbed Cd^2+^ were conducted using urea (0.1 M), HCl (0.1 M), EDTA (0.1 M) and NaCl (0.1 M). As shown in Figure 7a, the results revealed the superior effectiveness of HCl to recover Cd^2+^ from PVA-CDs, consistent with a previous study [84].

Next, regeneration tests were carried out using HCl as an eluent. As presented in Figure 7b, there is no substantial loss of % adsorption even after five adsorption-desorption cycles, in which only a 19.2% decline in the removal efficiency of the recycled material was shown. The loss in % adsorption might be assigned to the reduction of the adsorbent or the irreversible occupation of partial adsorption sites [85]. The good reusability emphasized that the adsorbent could be potentially used for practical contaminated water remediation.

### 2.10. Analysis of Real Water Samples

To further validate the applicability of the developed film, the removal of cadmium was tested in real water samples, including tap water and drinking water. The water samples were acquired from a biochemistry lab (UPM, Malaysia) and spiked with 50 µM Cd^2+^ ion solution. The values obtained were 95.34% and 90.9% in the case of tap and drinking water, respectively (Figure 7c). The results were compared with the same concentrations in distilled water with a % removal of 93.2%. These above data emphasized the reliability of the developed luminescence film for the recognition of Cd^2+^ in the environment.

## 3. Materials and Methods

### 3.1. Materials

CMC of oil palms empty fruit bunch wastes was collected from the Waris Nove Company (Pahang, Malaysia). Linear PEI (Mn ~5000) and quinine sulfate (quinine hemisulfate salt monohydrate) were purchased from Sigma-Aldrich (St. Louis, MO, USA). PVA (98% hydrolyzed, average molecular weight of 72,000 gmol^−1^) was obtained from R&M Chemicals (Selangor, Malaysia). Deionized water (DI) has been used throughout all the experiments.

### 3.2. Preparation of CDs and PVA-CDs

CMC (1 g) was mixed with PEI (1 g) and then dispersed in DI (250 mL). Then, the mixture was heated in an autoclave at 260 °C for 2 h. The resultant supernatant was centrifuged at 12,000 rpm for 10 min to obtain free-dark carbonaceous material. The final suspension was purified through vacuum filtration (0.22 µm) to remove the precipitate. It was then placed into a dialysis membrane (1 kDa) so that the contents of salt ions were eliminated. Polyvinyl alcohol-CDs film (PVA-CDs film) was prepared by mixing N-CDs (1 mL, 1 mg/mL) with PVA solution (1 mL, 1 wt%) under continuous stirring. Then, the obtained hydrogel was poured on a glass substrate and dried at 80 °C for 1 h. Finally, the PVA-CDs composite film was peeled off from the glass surface to obtain a freestanding film and stored for further utilization.

### 3.3. Characterizations

Transmission electron microscopy (TEM) and high-resolution TEM (HRTEM) readings were carried out using a G2 F20 electron microscope (Tecnai, Hillsboro, OR, USA) with an acceleration voltage of 200 kV. X-ray powder diffraction (XRD) patterns were measured using a PANalytical (Malvern, UK) diffractometer with Cu-Kα radiation. Fourier transform infrared (FTIR) spectra (Thermo Nicolet, Midland, ON, Canada) were measured of 4 cm^−1^ resolution with KBr as a standard within the range from 650 to 4000 cm^−1^. X-ray photoelectron spectra (XPS) (Physical Electronics PHI 5400 spectrometer, Mimos Semiconductors, Kuala Lumpur, Malaysia) were measured using Al-Ka radiation (hν=1486.6 eV). Before deconvolution, charge correction was established using Origin 9.0 (OriginLab Corporation, Northampton, MA, USA) at C_1s_ by setting binding energies of C-C and C-H at 284.8 eV. The pH values were obtained by a PB-10 pH-meter (Beijing Sartorius Instruments Co. Ltd., Beijing, China) and zeta potential study of N-CDs was carried out using Zetasizer Nano ZS (Malvern, UK). UV–Vis spectra of all samples were recorded using a UV-1800 spectrophotometer (Shimadzu, BA29AP, Corston, UK). Fluorescence spectra were measured in quartz cuvettes with 1 cm path length using a LS 55 Fluorescence Spectrometer (PerkinElmer, South San Francisco, CA, USA) with a slit width of 15 and 5 nm for excitation and emission, respectively, and a scan rate of 240 nm/min.

### 3.4. Quantum Yield Determinations

Quinine sulfate in 0.1 M H_2_SO_4_ (QY = 54%) was used as a standard fluorophore. The QY was determined by comparing both absorption and PL emission of the prepared and used materials with that of quinine sulfate using the following Equation (5) [48]:

(5) $$\mathrm{QY}=Q_{R}\;\frac{I}{I_{S}}\;\frac{OD_{S}}{OD}\;\frac{\eta^{2}}{\eta_{S^{2}}}$$

where (*I*) is integrated intensity, (OD) optical density and (η) is refractive index. The subscript S refers to the standard fluorophore of known QY.

### 3.5. Adsorption Studies

The feasibility of using the developed PVA–CDs films for heavy metal adsorption was evaluated. To test the selectivity, the hybrid films (0.1 g) were immersed in solutions of five selected metals: NiCl_2_, Pb(NO_3_)^2^, Cd(NO_3_)^2^, Zn(NO_3_)^2^, HgCl_2_ (30 mL, 50 ppm) at ambient temperature, pH = 7, then washed several times with DI and the supernatant then analyzed using atomic absorption spectrometry (AAS) to calculate the remaining metal ion in the solutions.

The kinetic tests were conducted under optimum conditions, agitating at 200 rpm. After a certain time (t) of adsorption, the residual Cd^2+^ concentration was measured using the aforementioned technique. For isotherm modeling, experiments were carried out at room temperature using various Cd^2+^ concentrations under optimal pH and contact time conditions. All the experiments were repeated three times. The equilibrium adsorption capacity, qe (mg/g) and removal efficiency, %adsorption was calculated using the following equations:

(6) $$qe=\frac{\left(Co-Ce\right)v}{m}$$

(7) $$\%\mathrm{Adsorption}\;=\frac{Co-Ce}{Co}\times 100$$

where *m* (g) is the mass of adsorbent, *v* (L) is the volume of solution, *Co* and *Ce* (mg/ L) are the initial and equilibrium concentration of Cd^2+^ in solution, correspondingly.

## 4. Conclusions

Eco-friendly photoluminescent CDs were produced from CMC and PEI. The as-made CDs solution with an abundance with oxygen and nitrogen species displayed a high quantum yield for monodispersed particles with a great graphitization index. The CDs were integrated with PVA to develop PVA-CDs fluorescent film which was utilized for the removal of Cd^2+^ from aqueous media. The results show that the film exhibited a bright blue color, a higher QY of 47% and an improved Cd^2+^ removal percentage of 91.1 % compared to that of bare CDs (greenish, 44%, 80.4%, respectively). The equilibrium adsorption time was 10 and 30 min for PVA-CDs and CDs, respectively, at an optimal solution pH of 7. The adsorption process could be explained by pseudo-second-order kinetics and the Langmuir isotherm model. Under maximum conditions, an optimal adsorption uptake of 113.6 mg g^−1^ was achieved. Nanocomplexation is proposed to be the main reason for the removal of Cd^2+^. The luminescent film was reusable for multiple desorption-adsorption cycles when regenerated with HCl solution. The feasibility of using the film was also tested for tap and drinking water with sufficient removal efficiencies. This work provides insights for developing an eco-friendly and rapid Cd^2+^ removal method with practical adsorbent membranes using sustainable and cost-effective carbon dots.

## Figures and Tables

**Table 1:** Kinetic models and isotherms parameters for Cd^2+^ adsorption onto PVA-CDs.

Pseudo-First Order			Pseudo-Second Order			Langmuir			Freundlich		
*K*_1_	*q_e_*	*R* ^2^	*q_e_*	*K_L_*	*R* ^2^	*q_e_*	*K_L_*	*R* ^2^	*K_f_*	*n*	*R* ^2^
0.1	5.4	0.9853	13.2	0.075	0.9961	113.6	0.016	0.9998	4.64	3.1	0.9853

**Table 2:** Comparison of several nanoadsorbents used for Cd^2+^ removal.

Adsorbent	Time (min)	*q_m_* (mg/g)	Ref.
NiFe_2_O_4_/hydroxyapatite/graphene quantum dots	10	344.83	[1]
Chitosan-carbon dots	3	112.4	[4]
Poly (acrylic acid)-activated carbon nanocomposite	120	473.2	[7]
SnO_2_ quantum dots decorated reduced graphene oxide	20	40.81	[46]
Carbon dots modified mesoporous organosilica	500	105.5	[44]
Carbon quantum dots/layered double hydroxide hybrid	20	12.6	[47]
Hollow calcite single crystals with CQDs	90	66.68	[45]
Multiwall carbon nanotubes	120	88.62	[72]
Nano-pumice	120	200	[73]
Nanoscale carbon black	120	31.7	[74]
N doped carbon dots integrated with PVA films	10	113.6	This work

## Supplementary Materials

The following are available online, Figure S1: Zeta potential of CDs, Figure S2: XPS spectrum of CMC, CDs and PVA-CDs, Figure S3: Photostability of PVA-CDs composite film at different aging times (a) and at different heating temperatures (b), Figure S4: Selectivity removal of Ni(II), Pb(II), Cd(II), Zn(II) and Hg(II) by using PVA-CDs composite film, Figure S5: Pseudo-first-order model (a) and Freundlich isotherm (b) for removal of Cd2+ onto PVA-CDs, Figure S6: (a) TEM image, (b) Zeta potential and (c) FTIR of PVA-CDs film in the presence of Cd2+. Inset: size distribution, Table S1: Elemental compositions of the EFB, CDs and PVA-CDs samples by XPS analysis.
