# Supplementary material for: Sustainable Development of Enhanced Luminescence Polymer-Carbon Dots Composite Film for Rapid Cd2+ Removal from Wastewater
Source: Molecules. 2020 Aug 3;25(15):3541. doi: 10.3390/molecules25153541 (PMC7436165; doi:10.3390/molecules25153541)
Supplement: Supplementary file 1 [file molecules-25-03541-s001.pdf]

molecules

MDPI

Supplementary Materials

Sustainable Development of Enhanced Luminescence Polymer-Carbon Dots Composite Film for Rapid Cd^{2+} Removal from Wastewater

Mohammed Abdullah Issa * and Zurina Z. Abidin *

Department of Chemical and Environmental Engineering, Faculty of Engineering, University Putra Malaysia, 43400 UPM Serdang, Selangor, Malaysia

* Correspondence: mohbaghdadi1@yahoo.com (M.A.I.), zurina@upm.edu.my (Z.Z.A.)

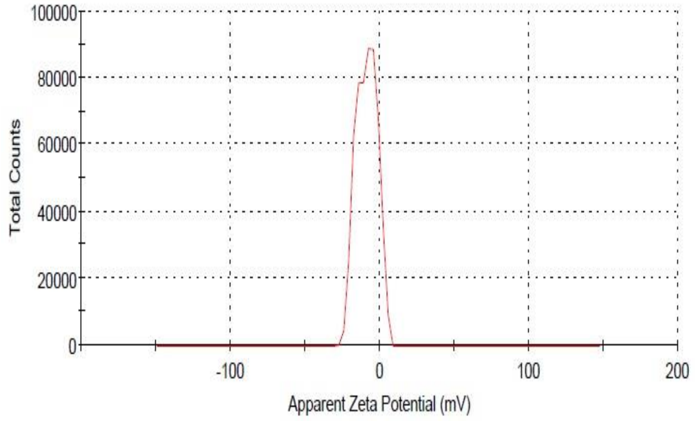

Figure S1. Zeta potential of CDs.

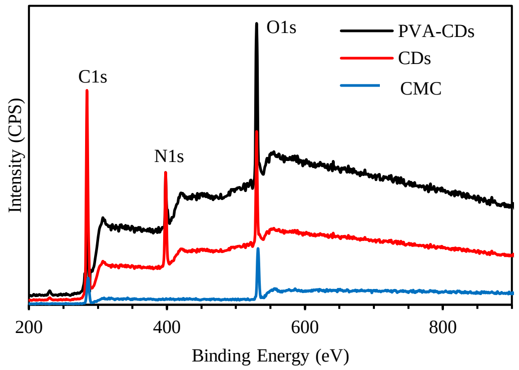

Figure S2. XPS spectrum of CMC, CDs and PVA-CDs.

Table S1. Elemental compositions of the EFB, CDs and PVA-CDs samples by XPS analysis.

**Sample**	**C (%)**	**O (%)**	**N (%)**	**Na (%)**
EFB	39.6	59.4	-	0.82
CDs	66.5	12.3	21.4	-
PVA-CDs	24.2	64.3	11.2	0.24

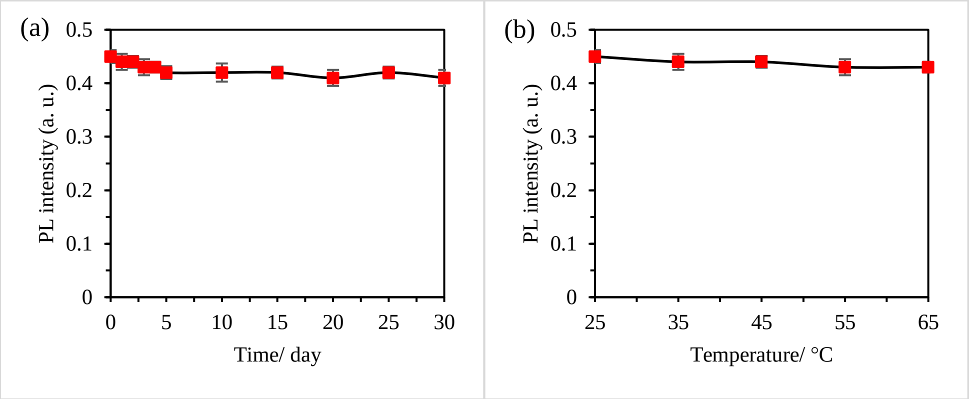

Figure S3. Photostability of PVA-CDs composite film at different aging times (**a**) and at different heating temperatures (**b**).

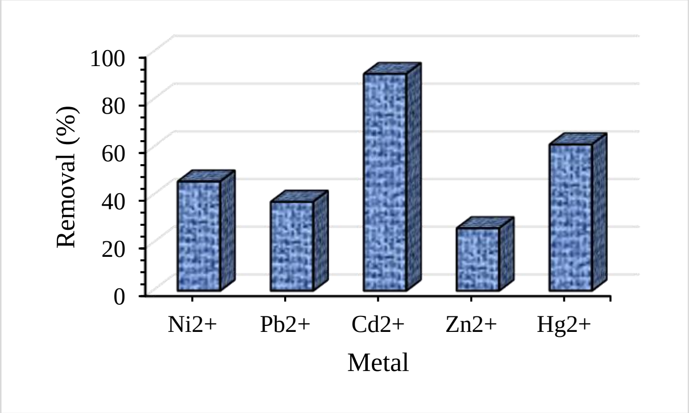

Figure S4. Selectivity removal of Ni(II), Pb(II), Cd(II), Zn(II) and Hg(II) by using PVA-CDs composite film.

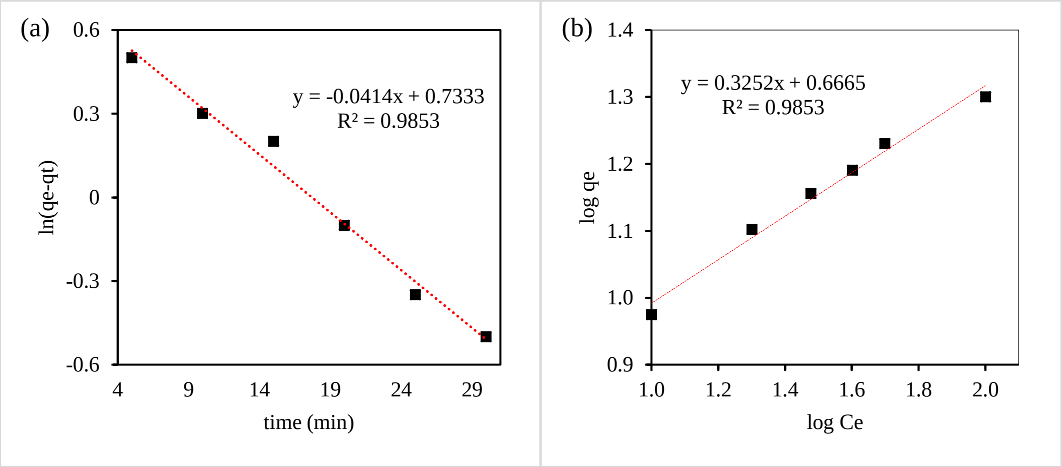

Figure S5. Pseudo-first-order model (a) and Freundlich isotherm (b) for removal of Cd^{2+} onto PVA-CDs.

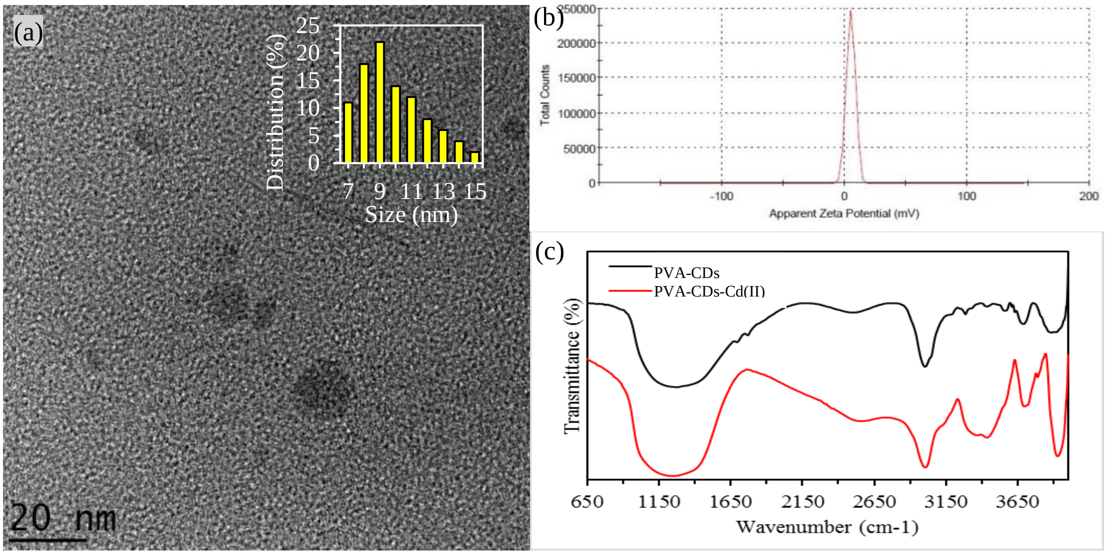

Figure S6. (**a**) TEM image, (**b**) Zeta potential and (**c**) FTIR of PVA-CDs film in the presence of Cd2+. Inset: size distribution.
